# Active Dry Yeast and Thiamine in Synergistic Mode Can Mitigate Adverse Effects of In Vitro Ruminal Acidosis Model of Goats

**DOI:** 10.3390/ani12182333

**Published:** 2022-09-08

**Authors:** Gulzar Ahmed, Hongrong Wang

**Affiliations:** 1 Laboratory of Metabolic Manipulation of Herbivorous Animal Nutrition, College of Animal Science and Technology, Yangzhou University, Yangzhou 225009, China; 2 Department of Livestock and Poultry Production, Faculty of Veterinary and Animal Sciences, University of Poonch Rawalakot, Rawalakot 12350, Pakistan

**Keywords:** active dry yeast, ruminal acidosis, lipopolysaccharides, microbial community, thiamine

## Abstract

**Simple Summary:**

Subacute ruminal acidosis (SARA) is a most prevalent metabolic disorder of ruminants which poses a great threat to the health and wellbeing of animals. The purpose of this research was to induce SARA in in vitro conditions and to determine the potential of active dry yeast (ADT) and thiamine synergistically in mitigating the adverse effects of SARA. Both the supplements ADY and thiamine synergistically enhanced the ruminal pH, decreased the abundance of negative rumen bacteria and increased the abundance of useful rumen bacteria and protozoa. It was concluded that the combined use of ADY and thiamine as a supplement could mitigate SARA. SARA not only damages the health of the animals but also has detrimental effects on the economic conditions of the farmers. Therefore, this research could be beneficial for society.

**Abstract:**

Ruminal acidosis is a type of metabolic disorder of high-yielding ruminants which is associated with the consumption of a high-grain diet. It not only harms the productive efficiency, health and wellbeing of the animals but also has detrimental effects on the economy of the farmers. Various strategies have been adapted to control ruminal acidosis. However, none of them have produced the desired results. This research was carried out to investigate the potential of active dry yeast (ADY) and thiamine in a synergistic mode to mitigate in vitro-induced ruminal acidosis. The purpose of this study was to determine how active dry yeast alone and in combination with thiamine affected the ruminal pH, lactate, volatile fatty acids, lipopolysaccharides (LPS) and microbial community in in vitro-induced ruminal acidosis. The experiment comprises three treatment groups, (1) SARA/control, (2) ADY and (3) ADYT (ADY + thiamine). In vitro batch fermentation was conducted for 24 h. The results indicated that ruminal induced successfully and both additives improved the final pH (*p* < 0.01) and decreased the LPS and lactate (*p* < 0.01) level as compared to the SARA group. However, the ADYT group decreased the level of lactate below 0.5 mmol/L. Concomitant to fermentation indicators, both the treatment groups decreased (*p* < 0.05) the abundance of lactate-producing bacteria while enhancing (*p* < 0.01) the abundance of lactate-utilizing bacteria. However, ADYT also increased (*p* < 0.05) the abundance of protozoa compared to the SARA and ADY group. Therefore, it can be concluded that ADY and thiamine in synergistic mode could be a better strategy in combating the adverse effects of subacute ruminal acidosis.

## 1. Introduction

In current farming systems, ruminants with higher production potential are usually offered a diet which is rich in concentrates and low in forages to enhance their production and efficiency. However, such kinds of feeding practices may increase the risk of metabolic disorders like subacute ruminal acidosis (SARA). SARA affects the gastric health and welfare of ruminants. Low rumen pH values (5.8–5.0) more than 3 h in a day is considered as the most significant indicator of SARA that takes place either repeatedly or which continues for a considerable amount of time [1]. In addition to ruminal volatile fatty acids (VFA), various other toxic compounds contribute to SARA’s pathophysiology. These toxic factors are biogenic amines like histamine [2] and lipopolysaccharide (LPS). These compounds are microbial in origin. LPS is a part of the Gram-negative bacteria’s cell wall, and is produced during the lysis of the bacteria. In contrast, biogenic amines are produced by decarboxylating the precursor amino acids under the degradation of ruminal microbes [3]. Therefore, sub-acute ruminal acidosis causes dysbiosis of ruminal microbiota and metabolic disorders [4]. Various strategies have been adapted to prevent sub-acute ruminal acidosis like the addition of sodium bicarbonate to the ration, or probiotics such as *Megasphaera elsdenii* as well as essential oils (cinnamaldehyde and eugenol) [5]. Active dry yeast and vitamin B1 also showed some good results in counteracting some adverse effects of SARA [6,7]. However, none of the strategies controlled SARA successfully The current study was designed to evaluate the potential of thiamine and active dry yeast in a synergistic mode against in vitro-induced ruminal acidosis. Therefore, the first objective of this research was to induce an in vitro model of ruminal acidosis in goats and the second object was to assess the modulating effects of active dry yeast alone and in combination with vitamin B1 on rumen fermentation indices and the microbial community in this ruminal acidosis model.

## 2. Materials and Methods

### 2.1. Donor Animals

Three rumen-cannulated dairy goats (body weight (BW): 38 ± 0.52 kg, mean ± SD) were utilized in this experiment as donors of rumen fluid. All the goats were offered a diet made up of 0.5 kg of concentrate (corn grain) and 0.5 kg of alfalfa hay. The Yangzhou University Animal Care and Use Committee granted approval for all experimental procedures to be conducted. Before the morning feeding, the rumen fluid was emptied into a container with a CO_2_-filled headspace after being filtered through four layers of muslin gauze from the goats’ fistulas. For 30 min at 39 °C, the container was left undisturbed, allowing the feed particles to float to the top of the bottle. The granules floating on the surface were removed, and the remaining fluid was used as inoculum.

### 2.2. Experimental Design and Sampling

The substrate (1 gm) made up of corn (500 mg), ground soymeal bean (150 mg), oat grass hay (150 mg) and alfalfa hay (200 mg) was dried in an oven, weighed and incorporated in a single bottle. The total 45 bottles were filled with the substrate and divided into 3 groups with groups (1) SARA/control group (Substrate + 0 additive) and two treatment groups, (2) ADY (Substrate 0.5 mg ADY) and (3) ADYT (Substrate + 0.5 mg ADY + 0.2 mg Thiamine)/bottle. The recommended dose of active dry yeast is 0.5 g/1 kg DMI/day providing 1× 10^4^ CFU of S. cerevisiae (ADY; Yea-Sacc^®^1026; Alltech, Nicholasville, KY, USA). As the substrate weight was 1 g, we incorporated 0.5 mg of active dry yeast into the treatment group. Similarly, the thiamine dose was calculated as 0.2 mg in the treatment group on the basis of a previous study done by Zhang et al., 2019 [8].

In every bottle, 8 mL of filtered ruminal fluid and 32 mL of 37 °C reduced buffer was incorporated [9]. After that, each bottle was flushed with CO_2_, closed and fermented at 39 °C for 24 h. For the analysis of rumen pH, lactic acid, NH3-N and microbial protein content (MCP), ruminal fluid was collected at 3 h, 6 h, 9 h, 12 h and 24 h of fermentation. For this, three bottles from each group at the designated time point were detached from the fermentation chamber. Thereafter, the termination of the fermentation process was carried out by shifting the fermentation bottles in an ice tub.

After the termination of incubation, the fluid pH was calculated by a portable pH meter (HI9024C, HANNA, Woonsocket, RI, USA). The incubated fluid was transported to centrifuge tubes, and the centrifugation was conducted at 12,000× *g* for 10 min to isolate the solid and liquid phases. Filtered liquid samples were preserved at −20 °C. Subsequently, the stored samples were used to evaluate lactic acid, volatile fatty acid (VFA), MCP, LPS and NH3-N concentrations. While solid models were converted into suspended solution by mixing them in the solution of phosphate buffer (PBS, pH 7.2) of 5 mL and preserved at −80 °C till it was used for the extraction of DNA.

### 2.3. Analysis of Fermentation Parameters

The concentration of VFA was measured by the gas chromatography (GC-14B; Shimadzu, Kyoto, Japan) technique proposed by Wang et al. [10]. Lactic acid and MCP were determined by following the methods proposed by Barker and Summerson (1941) [11] and Wang et al. [12], respectively, while NH3-N was measured by steam distillation method [13] and Keeney was determined by commercially available limulus amebocyte lysate assays (Xiamenhoushiji, Xiamen, China) as formerly revealed by Liu et al. [14].

### 2.4. Extraction of DNA and RT_PCR

Freeze-dried samples of the suspended solution were ground with the help of a pestle and mortar. Freeze drying has been considered beneficial in enhancing the quality and quantity of DNA [15]. DNA was extracted to follow the method of Malekkhahi et al., 2016 [14]. After the extraction of the DNA, the samples were stored at −80 °C until further investigation. Subsequently, the extracted DNA was served as a template of the polymerase chain reaction.

The relative abundance of *Prevotella albensis*, *Streptococcus bovis*, *M. elsdenii*, *Fibrobacter succinogenes*, *S. ruminantium*, *Anaerovibrio lipolytica*, *Lactobacillus* spp., *Ruminococcus albus* and *Protozoa* were analyzed by RT-PCR by following the procedure of a previous study [16]. Table 1 shows the primers of the microbial species. The primers were assembled from the literature to amplify bacteria 16S rRNA and protozoal 18S rRNA region. Real-time PCR was performed by employing an AB 7300 system (Applied Biosystems, Foster City, CA, USA). Each reaction mixture was run in twice. Power SYBR green PCR master mix (Applied Biosystems, Foster City, CA, USA) in combination with the designated primer set was used for the amplification of the reaction.

Changes in prescribed populations (fold changes) of *Prevotella albensis*, *Streptococcus bovis*, *M. elsdenii*, *Fibrobacter succinogenes*, *S. ruminantium*, *Anaerovibrio lipolytica*, *Lactobacillus* spp., *Ruminococcus albus* and *Protozoa* were determined employing a relative quantification calculation and the method [17], concerning the general bacteria [18] cycle threshold (CT) values used as the reference and average Ct of the CON group as the calibrator value.

### 2.5. Statistical Analysis

The statistical analysis of the data was done using SPSS 16.0 software (SPSS Inc., Chicago, IL, USA). A one-way ANOVA and Duncan’s post hoc multiple comparisons test were used to assess the data on the signs of fermentation as well as the abundance of the ruminal microbial community. At *p* < 0.05, a difference was deemed significant.

## 3. Results

### 3.1. Fermentation Parameters

Table 2 indicates that SARA is successfully induced. The ADYT supplementation prevents the reduction of pH value at the threshold level of 5.8. However, ADY also improved the rumen pH (*p* < 0.01) as compared to the SARA group. Table 3 shows that lactic acid concentrations in both the treatment groups decreased (*p* < 0.01) but the response of ADYT in the reduction of lactate was more obvious. Table 4 depicts that after 12 h and 24 h of fermentation, the concentration of NH3-N (*p* < 0.01) decreased in treatment groups compared to the SARA group. Table 5 represents that the concentration of MCP increased significantly after 24 h of incubation in ADYT group. Table 6 reflects that the concentration of TVFA, acetic acid and A/P significantly increased (*p* < 0.01) in treatment groups while the concentration of propionic acid and LPS decreased (*p* < 0.01) in treatment groups compared to the SARA.

### 3.2. Rumen Microbial Community

Table 7 illustrates the fold change in the abundance of microbial populations among the treatment groups. The results indicate that the abundance of *S. bovis*, *P. albensis* and *Lactobacilli* decreased (*p* < 0.05) in treatment groups compared to the SARA group. In contrary to this, the abundance of *Anaerovinriyplytic*, *M. elsdenii*, *Ruminococcus albus* and *S. ruminantium* was enhanced *p* < 0.05 in the treatment groups. However, the abundance of *F. succinogens* and protozoa in the SARA and ADY group significantly decreased (*p* < 0.05) compared to the ADYT group.

## 4. Discussion

### 4.1. Fermentation

The fermentation of a high-concentrate diet often results in the production and accumulation of a higher amount of lactic acid in the rumen [8,24]. The mechanism of controlling ruminal acidosis is to inhibit lactate-producing microbial communities and to stimulate the activity of lactate-utilizing bacteria or starch-engulfing protozoa [25]. We hypothesized that thiamine and active dry yeast synergistically alleviate in vitro-induced subacute ruminal acidosis by batch culture method. The alleviation was suggested to be achieved by increasing the pH level and reducing the content of lactic acid and other toxic compounds like lipopolysaccharide (LPS). In the current study, ruminal pH declined constantly from 0 h to 24 h of fermentation. The interesting point is that the pH declines in a similar pattern after the grain feeding in vivo studies in the SARA group [23,26]. Reduction in ruminal pH < 5.8 for more than 3 h/24 h is considered a threshold value for establishing SARA [27]. After 12 to 24 h of incubation, the pH in the SARA group was noted below the threshold value of 5.8 in the SARA/control group while in treatment 1 the pH value was above 5.8 and 5.6 at 12 h and 24 h of fermentation, respectively. While in treatment 2, the pH value was higher at 12 and 24 h of incubation. This shows that this may be due to the synergetic effect of ADY and thiamine. Contrary to our study, AlZahat et al. found that ADY supplementation did improve the rumen pH value significantly (*p* < 0.05) compared to the SARA [6]. This shows that active dry yeast can stabilize rumen pH more effectively in combination with thiamine. Although some studies show that active dry yeast alone can stabilize the rumen pH during ruminal acidosis in ruminants [28], other studies show that active dry yeast has not affected the ruminal pH under high-grain feeding [29]. Thiamine supplementation under high-grain feeding has a positive correlation with rumen pH [30], and past studies show that inclusion of thiamine in a grain-rich diet can improve the ruminal pH [7].

As compared to the pH value, the concentration of lactic acid decreased at 12 and 24 h in treatment groups compared to SARA. The decrease in lactic acid concentration is associated with an increase in ruminal pH, and active dry yeast may stimulate the growth of cellulolytic bacteria. The results of our study are consistent with the task of Lilla et al. [31]. Inclusion of thiamine in grain-rich diet favors the growth of lactate-utilizing bacteria [23], and similar to our findings, Pan et al.,2016 found that the lactic acid concentration was low in the treatment group compared to the SARA group [11]. Contrary to our study, active dry diet supplementation did not show any difference between the SARA and treatment groups [10,14].

In the SARA group, the concentrations of LPS in our study was higher as compared to findings Yin et al. [32] in in vitro-induced ruminal acidosis model. LPS is an endotoxin which is the part of Gram-negative bacteria. It releases in the rumen due to lysis of the bacteria during ruminal acidosis as these bacteria are pH-sensitive [33]. Previous studies indicated that active dry yeast alone or in combination with other additives could not minimize LPS in high-grain-fed sheep [34].

Contrary to that, LPS level significantly decreased in both treatment groups. Supplementation of active dry yeast improves the rumen environment by making it more conducive for the growth of fibro lytic bacteria by removing oxygen from it [6,35]. As ruminal acidosis causes an imbalance between lactate-producing and lactate-utilizing bacteria and inclusion of thiamine can facilitate a balance between them [23] and reduces LPS concentration in SARA-induced goats and cattle [7,8].

The disturbance in the microbial community as a result of grain feeding plays a vital part in the etiopathology of sub-acute ruminal acidosis [36]. Hence, the response of ADY in combination with thiamine is more promising in the stabilization of fermentation indicators.

### 4.2. Bacterial Community

It is evident from the results that as compared to ADY, ADYT supplementation in in vitro rumen acidosis could improve rumen function by enhancing ruminal pH and acetic acid and reducing lactic acid and LPS in a better way. This progress in rumen fermentation indicators could be the reflection of the improved microbial community [37]. In keeping view of it, we further investigated the microbial response to the SARA group and both treatment groups.

The inclusion of thiamine in a high-grain diet improves the growth of cellulytic bacteria [38].

The population of cellulytic bacteria like F. succinogenes lowered during the subacute ruminal acidosis challenge [39]. Saccharomyces cerevisiae promotes the growth of F. succinogenes and other fibrolytic bacteria [28]. The impact of ADY on the abundance of F. succinogenes agrees with previous studies [29,40] where supplementation of ADY did not improve the growth of the bacteria. But in combination with thiamine, it enhanced the bacterial population as compared to the SARA group. This could be due to the higher pH value in the ADYT group.

Under lower ruminal pH, lactate concentration decreases [41] because the abundance of lactate-utilizing bacteria like *Megasphaera elsdenii* start declining during high-grain feeding [23]. ADY can enhance ruminal pH by improving lactate utilization [42] through facilitating the growth of *Megasphaera elsdenii* and *selemonas* [37]. However, in our study, the ADYT group significantly enhanced the abundance of *Megasphaera elsdenii* and *selemonas* while ADY effected positively on the growth of *Megasphaera elsdenii*. This is consistent with the findings of Chaucheyras-Durand et al., 2008. Contrary to our study, the ADY supplementation showed no effect against *Megasphaera elsdenii* in SARA-induced dairy cattle [29].

*S. bovis* and *P. albensis* are a Gram-negative bacterium which prevails in a high concentration during ruminal acidosis and considered a key source of LPS [36]. LPS may damage the health of affected animals like laminitis [43]. The ADYT group significantly depicted a reduction in the abundance of *P. albensis* compared to ADY. This reflects that ADYT supplementation could help in minimizing the negative consequences of ruminal acidosis on the health of animals.

### 4.3. Protozoa

Protozoa are sensitive to low ruminal pH, in an intensive farming system, grain feeding is a common practice which may imbalance the ruminal protozoa. The decline protozoa in the SARA group is in line with the study of Owens et al. (1998), who established that fibrous mat is essential for the multiplication of protozoa [44]. Slyter, 2004, indicated that high osmotic pressure along with low ruminal pH could have a direct lethal effect on protozoa [44]. The result indicated that ADY supplementation did not affect protozoa. This agrees with the study of AlZahal et al., 2014, where ADY supplementation could not enhance protozoa in SARA-induced cattle [6]. However, ADYT showed a positive effect (*p* < 0.05) on protozoa. The better protozoa population detected with ADYT than ADY and the SARA group is perhaps directly related to the increased pH values in the ADYT group.

## 5. Conclusions

It is concluded that a substrate comprises 50% corn grain and 15% soybean meal is sufficient to induce subacute ruminal acidosis in in vitro conditions. The results further confirmed that both the treatment groups improved ruminal pH but as compared to the ADY group, the ADYT group showed a significant reduction in the abundance of lactate-producing bacteria, lactate and LPS content and increased the abundance of lactate-utilizing bacteria and protozoa. Therefore, supplementation of ADY and thiamine in combination would have the potential to mitigate sub-acute ruminal acidosis. Further, in vivo studies can be recommended to validate the results of the in vitro study.

## Figures and Tables

**Table 1:** Real-time PCR primers are used to amplify DNA.

Target Organism	Forward/Reverse	Primer Sequence	Product Size	References
General bacteria	F R	GTGSTGCAYGGYTGTCGTCA ACGTCRTCCMCACCTTC	146	[19]
*S. bovis*	F R	TTCCTAGAGATAGGAAGTTTCTTCGG ATGATGGCAACTAACAATAGGGGT	127	[20]
*Prevotella albensis*	F R	GCGCCACTGACGCTGAAG CCCCAAATCCAAAAGGACTCAG	110	[21]
*F. succinogenes*	F R	F GTTCGGAATTACTGGGCGTAAA CGCCTGCCCCTGAACTATC	121	[22]
*M. elsdenii*	F R	AGATGGGGACAACAGCTGGA CGAAAGCTCCGAAGAGCCT	79	[20]
*S. ruminantium*	F R	CAATAAGCATTCCGCCTGGG TTCACTCAATGTCAAGCCCTGG	71	[15]
*Lactobacillus*	F R	AGCGAACAGGATTAGATACCC GATGGCACTAGATGTCAAGACC	233	[23]
*Anaerovibrio lipolytica*	F R	TGGGTGTTAGAAATGGATTCTAGTG GCACGTCATTCGGTATTAGCAT	109	[21]
*Ruminococcus albus*	F R	CCCTAAAAGCAGTCTTAGTTCG CCTCCTTGCGGTTAGAACA	176	[6]
Protozoa	F R	GCTTTCGWTGGTAGTGTATT CTTGCCCTCYAATCGTWCT	223	[18]

**Table 2:** Effect of ADY and ADYT on pH value at different time points of incubation.

pH Noted at Hours of Incubation	SARA/Control	ADY	ADYT	SEM	*p*-Value
0 h	6.58	6.56	6.56	0.001	0.23
3 h	6.43	6.44	6.46	0.014	0.17
6 h	6.25 b	6.27 b	6.37 a	0.021	0.004
9 h	5.95 c	6.05 b	6.22 a	0.021	0.00
12 h	5.68 c	5.81 b	6.04 a	0.027	0.00
24 h	5.56 c	5.70 b	5.92 a	0.021	0.00

Subacute ruminal acidosis. Active dry yeast. Active dry yeast + Thiamine. a–c Means denoted by various superscripts differ significantly (*p* < 0.05).

**Table 3:** Effect of ADY and ADYT on Lactic Acid.

Lactic Acid (mmol/L) at Various Time Points Incubation	SARA/Control	ADY	ADYT	SEM	*p*-Value
3 h	0.28	0.27	0.27	0.01	0.85
6 h	0.34	0.33	0.33	0.00	0.35
9 h	0.39	0.38	0.36	0.011	1.60
12 h	0.54 a	0.44 b	0.41 b	0.024	0.003
24 h	0.76 a	0.53 b	0.45 c	0.028	0.00

a–c Means denoted by various superscripts differ significantly (*p* < 0.05).

**Table 4:** Effect of ADY and ADYT on NH3-N.

NH3-N (mg/dL) at Different Hours of Incubation	SARA/Control	ADY	ADYT	SEM	*p*-Value
3 h	18.16	18.30	18.54	1.005	0.932
6 h	22.22	22.43	21.88	1.45	0.930
9 h	25.61	23.44	24.47	1.25	0.297
12 h	33.79 a	30.33 b	29.28 b	1.34	0.035
24 h	27.99 a	22.52 b	21.23 b	1.37	0.06

a, b Means denoted by various superscripts differ significantly (*p* < 0.05).

**Table 5:** Effect of ADY and ADYT on Microbial protein content (MCP).

MCP (mg/mL) at Different Hours of Incubation	SARA/Control	ADY	ADYT	SEM	*p*-Value
3 h	5.41	18.30	18.54	0.71	0.849
6 h	5.59	22.43	21.88	0.070	0.697
9 h	5.69	23.44	24.47	0.089	0.56
12 h	5.88	30.33	29.28	0.073	0.508
24 h	6.25 b	6.35 ab	6.43 a	0.050	0.30

MCP = microbial protein. a, b Means denoted by various superscripts differ significantly (*p* < 0.05).

**Table 6:** Effect of ADY and ADYT on Volatile fatty acids and Lipopolysaccharide.

Items	SARA/Control	ADY	ADYT	SEM	*p*-Value
TVFA (mmol/L)	113.03 b	124.27 ab	132.91 a	6.40	0.056
Acetic Acid (%)	51.91 c	56.15 b	61.16 a	1.41	0.002
Propionic Acid (%)	28.65 a	23.34 b	21.23 b	1.003	0.001
Butyric Acid (%)	15.85	16.51	13.50	1.57	0.216
Isobutyric Acid (%)	0.68 c	0.88 b	1.43 a	0.035	0.000
Isovaleric Acid (%)	1.52 a	1.35 b	1.54 a	0.068	0.061
Valeric Acid%	1.39 b	1.77 a	1.25 c	0.039	0.001
A/P	1.81 c	2.40 b	2.89 a	0.085	0.002
LPS (EU/mL)	17,857.81 a	13,051.27 b	9875.32 c	1211.23	0.002

TVFA = acetate + propionate + butyrate + valerate + isobutyrate + isovalerate. LPS = Lipopolysaccharide. A/P = Acetate to propionate ratio. a–c Means denoted by various superscripts differ significantly (*p* < 0.05).

**Table 7:** Effect of ADY and ADYT on the abundance of rumen microbial community.

Items	SARA/Control	ADY	ADYT	SEM	*p*-Value
*S. bovis*	1.00 a	0.833 a	0.59 b	0.07	0.003
*P. albensis*	1.00 a	0.72 b	0.53 c	0.058	0.001
*Lactobaciilli*	1.00 a	0.78 b	0.44 c	0.082	0.001
*Anaerobic lyplitica*	1.00 b	1.23 b	1.62 a	0.096	0.002
*M. elsdenii*	1.00 b	1.20 b	1.65 a	0.132	0.007
*R. albus*	1.00 b	1.43 b	1.76 a	0.142	0.005
*S. ruminantium*	1.00 b	1.17 b	1.61 a	0.084	0.001
*F. succinogenes*	1.00 c	1.24 b	1.76 a	0.1150	0.002
Protozoa	1.00 b	1.12 b	1.77 a	0.098	0.007

a–c Means denoted by various superscripts differ significantly (*p* < 0.05).

## Data Availability

This article contains all the data that were created or examined during this investigation.
